# Serum 25-Hydroxy Vitamin D in Chronic Rhinosinusitis with and Without Nasal Polyposis: A Case-Control Study 

**Published:** 2019-01

**Authors:** Ali Faghih Habibi, Hooshang Gerami, Rahmatollah Banan, Ehsan Kazemnezhad Leily, Parsa Khoshkhat, Hooman Alaie Alamouti, Shadman Nemati

**Affiliations:** 1 *Rhino-Sinus, Ear, and Skull base Diseases Research Center, Department of Otolaryngology and Head and Neck Surgury, Amiralmomenin Hospital, School of Medicine, Guilan University of Medical Sciences, Rasht, Iran.*; 2 *International Campus, School of Pharmacy, Tehran University of Medical Sciences, Tehran, Iran.*

**Keywords:** Chronic, Nasal Polyps, Rhinitis, Sinusitis, Vitamin D

## Abstract

**Introduction:**

Chronic rhinosinusitis (CRS) with and without nasal polyposis is a chronic inflammatory disease of the sinuses and nasal mucosa. Recent evidence has indicated a relationship between serum 25-hydroxyl vitamin D (OH-VitD) deficiency and CRS. Regarding this, the present study aimed to compare the serum level of 25-OH-VitD in CRS patients with and without nasal polyposis and control groups.

**Materials and Methods:**

This study was conducted on 117 adult subjects in three groups of CRS with nasal polyposis (CRSwNP; n=32), CRS without nasal polyposis (CRSsNP; n=35), and healthy controls (n=50). The mean level of serum 25-OH-VitD in the three groups was measured by means of enzyme- linked immunosorbent assay. The data were analyzed using SPSS software (version 18).

**Results:**

Mean serum levels of 25-OH-VitD in CRSwNP, CRSsNP, and control groups were 12.52, 15.54, and 22.04 ng/ml, respectively. There was a significant difference between the case and control groups in terms of 25-OH-VitD level (P=0.0001). However, no significant difference was observed between the CRSwNP and CRSsNP groups in this regard (P=0.464). The women had a VitD deficiency odds ratio (OR) of 2.47, compared with men (OR=2.47, 95% CI=1.04-5.86). The OR of VitD deficiency with aging was obtained as 0.957 (95% CI=0.925-0.989). In this regard, older patients had a lower probability of VitD deficiency, compared to younger patients.

**Conclusion:**

As the findings indicated, serum 25-OH-VitD was significantly lower in CRS patients, compared with that in the non-CRS subjects.

## Introduction

Chronic rhinosinusitis (CRS) is an inflammatory disease of nasal mucosa and sinuses that is clinically classified into CRS with nasal polyposis (CRSwNP) and CRS without nasal polyposis (CRSsNP). The prevalence of this disease has been reported to range within 12-15% in different studies, which is relatively high among chronic inflammatory diseases (1). 

The CRS is a multifactorial disease with unknown etiologic and pathophysiologic aspects. The proposed etiologies for this disease include anatomic factors, infectious causes, fungal allergies, immunological disorders, biofilms, gastroesophageal reflux disease, and genetic causes (2,3). The final result of all these pathophysiological factors is a chronic inflammation in the sinonasal mucosa (2).

One of the recently proposed factors that may have some roles in the pathophysiology of CRS is 25-hydroxyl vitamin D (OH-VitD) deficiency. The role of VitD, which has a structure similar to that of steroids, has been suggested as an immunomodulator agent. Accordingly, the deficiency of this vitamin has been identified as an etiologic factor in many chronic inflammatory diseases, including inflammatory bowel disease, multiple sclerosis, allergic rhinitis, and asthma (4-6). Low serum 25-OH-VitD levels also have been related to airway hyperresponsiveness, impaired pulmonary function, increased frequency of asthma exacerbation, and decreased responsiveness to corticosteroids (7). 

The basic etiologies of CRS are unknown; however, it has been supposed that VitD intensifies the anti-inflammatory effects of corticosteroids in asthmatic cases by the enhancement of the ability of T cells to produce interleukine-10 or inhibition of cytokine production by T helper cell (T_H_17). Furthermore, deficient VitD levels can create a proinflammatory state; in this regard, VitD signaling pathways and receptor polymorphisms can affect the balance between T_H_1 and T_H_2 (7).

Some epidemiological studies have shown an association between deficient VitD levels and some respiratory tract infections (e.g., tuberculosis) (8). Vitamin D has a role in immune responses, including cathelicidins induction (a group of antimicrobial peptides produced by neutrophils, macrophages, and epithelial cells) (9). Several studies have also reported an inverse relationship between serum 25-OH-Vit D levels and incidence of upper respiratory tract infection (8).

Considering CRS as a chronic inflammatory disease with immunological aspects, and given the histopathological similarities between CRS and asthma, it was proposed that VitD deficiency may have a role in the pathophysiology of CRS (10). With this background in mind, the present study aimed to investigate the relationship between the serum levels of 25-OH-VitD and presence of CRS for the first time among a group of Iranian subjects.

## Materials and Methods

This cross-sectional controlled study was conducted on people over 18 years of age referred to Amiralmomenin University Hospital in Guilan province, north of Iran, from June 2014 to May 2016. The proposal of the study was reviewed and approved by the Rhinosinusitis, Ear, and Skull Base Research Center Review Board and the Ethics Committee of Research and Technology Secretary of Guilan University of Medical Sciences, Guilan, Iran. Patients with the clinical evidence of CRS were enrolled in the study. The exclusion criteria were: 1) consumption of VitD supplements during the last 6 months, 2) long-term treatment with steroids (i.e., more than 1 month), 3) affliction with rickets, osteomalacia, cystic fibrosis, multiple sclerosis, rheumatoid arthritis, sarcoidosis, ulcerative colitis, Crohn's disease, and thyroid dysfunction, 4) consumption of sulfasalazine, bisphosphonates, or barbiturates, 5) pregnancy, and 6) history of prior sinus surgery.

Clinical diagnosis of CRS was based on the presence of two major criteria of CRS or one major and two minor criteria, and persistence of the signs and symptoms for more than 12 weeks (10). The major criteria were facial pressure or pain, facial fullness, nasal obstruction, nasal discharge or postnasal mucus, anosmia or hyposmia, and purulent discharge in rhinoscopy examination. On the other hand, the minor criteria entailed headache, fever, bad breath, fatigue, and pain or fullness in the jaw and ear. 

In all cases, disease diagnosis was confirmed by the observation of at least one objective modality, such as computed tomography (CT) scan and/or endoscopic examination of the nose and paranasal sinuses. Endoscopic evidence for CRS included the presence of polyps in the nasal middle meatus, discolored mucus, edema of the middle meatus or ethmoidal sinus mucosa, and purulent discharge in osteomeatal unit. Presence of polyps in the middle meatus along with the other criteria of CRS confirmed the diagnosis of CRSwNP, whereas its absence approved the diagnosis of CRSsNP (1). 

All Sinonasal endoscopies were performed by the same expert in the Rhinology Clinic of the hospital under investigation on an outpatient basis under topical anesthesia. The subjects of the control group were selected from the referred patients with no clinical evidence of CRS or healthy personnel of the hospital. The exclusion criteria in this group were similar to those considered for the case groups. Considering the exclusion criteria, all the control subjects were referred to rhinology clinic for undergoing sinonasal endoscopy in order to rule out CRS.After informing the case and control groups about the study objectives and obtaining their written informed consent, they were entered into the study. In three groups, sampling was performed through the random selection of the subjects using random block numbers. All the subjects in the case and control groups had Islamic-Iranian wearing style. Because of the probable effects of seasons on the serum level of VitD, sampling was performed throughout two consecutive years while the distribution of the subjects in each group was similar in each season. In order to measure the serum levels of 25-oH-VitD, 1.5 cc fasting blood was taken and kept in plain glass tubes to be sent for analysis by the enzyme-linked immunosorbent assay using Euroimmun kit (PerkinElmer company-Germany).

**Statistical analysis**

Statistical analysis was performed in SPSS software (version 18). Q-square and ANOVA tests were used to compare the gender and age distributions among the groups, respectively. Furthermore, Shapiro test was used for the evaluation of the normality of serum levels of VitD in three groups. The two- and three-group comparison of the mean serum levels of VitD was accomplished using ANOVA and Tukey’s post hoc test. P-value less than 0.05 was considered statistically significant.

## Results

A total of 117 subjects were enrolled in the study in three groups of CRSwNP (n=32; 17 females and 15 males), CRSsNP (n=35; 19 females and 16 males), and control (n=50; 26 females and 24 males) groups. The results of the Q-square test revealed no significant difference among the three groups in terms of gender distribution (P=0.97). The CRSwNP, CRSsNP, and control groups had the mean ages of 38.78, 37.94, and 42.2 years, respectively. Based on the results of the ANOVA test, there was no significant difference among the three groups regarding age (P=0.302).

The results of the Shapiro test showed the non-normality of VitD serum level measures; therefore, Mann-Whitney U and Kruskal Wallis tests were utilized for inter-group comparison. The mean serum VitD levels in the control, CRSwNP, and CRSsNP groups were obtained as 22.04, 12.52, and 15.54 ng/ml, respectively (range: 4.0-60.4, 4.0-45, and 4.0-49 ng/ml, respectively). The results revealed a significant difference among the three groups in terms of the serum levels of VitD (P=0.0001)(Table.1).

**Table 1:** Comparison of vitamin D serum levels among three study groups based on Kruskal Wallis test

	**Group size**	**Mean**	**Std. Deviation **	**Mean rank**	**Minimum**	**Maximum**	**P-value**
Control	50	22.04	10.92	74.77	4.00	60.40	0.0001
CRSwNP	32	12.52	9.93	40.77	4.00	45.00	
CRSsNP	35	15.54	10.09	53.14	4.00	49.00	
Total	117	17.49	11.11		4.00	60.40	

CRSwNP: chronic rhinosinusitis with nasal polyposis, CRSsNP: chronic rhinosinusitis without nasal polyposis

Mann-Whitney U test showed that the serum levels of VitD in both CRS groups (i.e., with and without polyposis) were significantly lower than that in the control group (P=0.015 and P=0.0001, respectively). However, there was no significant difference between the CRSwNP and CRSsNP groups in this regard (P=0.08).

In the multiple analysis of the relationship between CRSwNP and CRSsNP in terms of VitD serum level, using backward logistic regression (with the significance levels of 0.05 and 0.1 for entry and removal, respectively), VitD response below the mean (i.e., 17.5) was assumed as 1 and more than the mean was considered as 0. In the unadjusted model, relative odds ratios (ORs) in CRSwNP and CRSsNP groups were 7.7 and 3.4 times more than that of the control group (OR=7.7, 95% CI: 2.67-22.2 and OR=3.4, 95% CI: 1.37-8.43, respectively).

**Fig 1:**
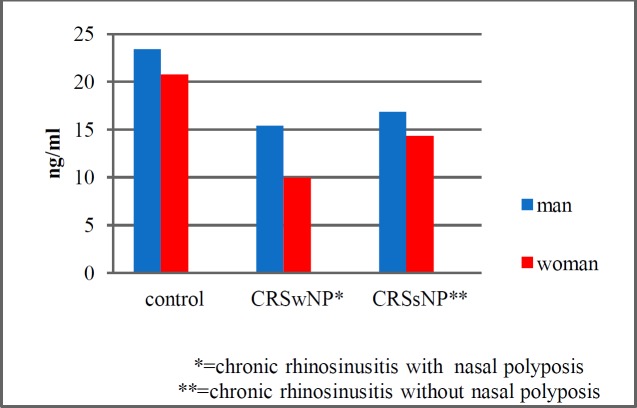
Serum levels of vitamin D in the case and control groups based on gender

In addition, in the adjusted model for age and gender, relative ORs in CRSwNP and CRSsNP were 8.7 and 3.1 times more, compared to that of the control group (OR=8.7, 95% CI: 2.7-27.7 and OR=3.4, 95% CI: 1.2-7.9, respectively). With increasing age, the OR of VitD deficiency was below 1 (OR=0.957, 95% CI: 0.925-0.989); in other words, older patients had a lower probability of VitD deficiency, compared with younger patients (P=0.01). Finally, the mean serum levels of VitD in the control group were 23.4 and 20.79 ng/ml among the males and females, respectively (P=0.41). These values were 15.4 and 9.98 ng/ml in the CRSwNP, and 16.87 and 14.32 ng/ml in CRSsNP groups, respectively (Fig.1). 

The OR of VitD deficiency in women was 2.47, compared that in men (OR=2.47, 95% CI: 1.04-5.86; P=0.04).

## Discussion

In the present study, the mean serum level of VitD in the control group was 22.04 ng/ml. In a study conducted in Tehran, Iran, (2004) on 1,210 healthy males and females, the mean serum level of VitD was reported as 20.65 ng/ml (11). In another study conducted in 2007 on 1,111 healthy participants in Isfahan, Iran, the mean levels of this vitamin were estimated as 18 and 21 ng/ml in females and males, respectively (12). The serum level of 25-OH-VitD in the mentioned studies was similar to that obtained in our study, although those studies had fewer exclusion criteria, compared with our study (11,12). 

In the current study, VitD serum levels were lower in women and in lower age groups than those in men and subjects of a higher age group. This is in contrast with the findings reported by Sherman et al. (13). However, the results of other studies performed on the relationship of age and gender with VitD serum level are inconsistent (13-15). Only two other studies carried out in Tehran and Isfahan showed higher levels of VitD in elderly females, which is incongruent with the results obtained in other studies. This discrepancy may be due to the consumption of supplements, such as multivitamins and compounds, affecting the serum level of VitD, by the subjects of those studies (11,12).

In the current study, the mean VitD serum level in the control group was 22.4 ng/ml, which was significantly higher than those of the CRSwNP (12.52ng/ml) and CRSsNP (15.54 ng/ml) groups. Although the mean serum levels of the CRSwNP group was lower than that of the CRSsNP, the difference was not statistically significant. 

In two similar studies performed by Apuhan et al. in Turkey and Mulligan et al. in the United States, serum VitD levels in patients with CRS and healthy control groups were compared (16,17). Apuhan et al. (2011) conducted their study on 20 controls and 40 patients suffering from CRSwNP. Like our study, in the mentioned study, CRS was diagnosed clinically and confirmed by the objective modalities, such as CT scan or nasal endoscopy; furthermore, serum VitD level was measured by ELISA. Nonetheless, their exclusion criteria were much fewer than those adopted in the present study. In the mentioned study, the level of VitD serum in the control and CRSwNP groups were reported as 24.62 and 24.95 ng/ml, respectively. In contrast to our study, there was no significant difference between the control and CRSwNP groups in terms of the VitD serum level. However, in line with our study, they observed no relationship between VitD serum levels and the age and gender (16). 

There are also inconsistent results; for example, in a study performed in Turkey in 2014, Bozkurt et al. studied VitD serum level in 440 healthy males and females and concluded that VitD serum level in females and males were 12 and 14.7 ng/ml, respectively (18). In a study carried out by Alagol et al. in Turkey (2000), the mean VitD serum level was estimated in 48 females within the age range of 14-43 years, depending on dressing style. In this regard, they reported the mean serum levels of 56.41, 31.9, and 9 ng/ml in the individuals dressing in the European, Islamic (the areas exposed to the sun are hands and face), and traditional Islamic (the face is also covered) styles, respectively (19). Different styles of wearing, from western to traditional Islamic, in Turkey can significantly affect the results of the mentioned study, such as the measurement of VitD serum levels. 

This issue was investigated in a study carried out by Guzel et al. in 2001 in Turkey (20). In the mentioned study, serum VitD levels were compared between 30 females with a western wearing style and 30 females with an Islamic wearing style. The VitD serum levels in the western and Islamic wearing style groups were reported as 53.9 and 33.1 ng/ml, respectively (20).

In a study conducted by Mulligan et al. ^17 ^in 2011 in the US, VitD serum levels were evaluated in 14 patients with allergic fungal rhinosinusitis (AFRS), 9 cases with CRSwNP, 40 patients with CRSsNP, and 14 patients hospitalized due to the cerebrospinal fluid  leak as the control group. In addition, the level of dendritic cells in the systemic circulation was studied using flow cytometry and immunoassay. Their results showed lower serum levels of VitD and higher levels of circulating dendritic cells in the patients with AFRS and CRSwNP, compared with those in the control group. 

Furthermore, they observed no difference between the CRSsNP and control groups regarding the serum levels of 25-OH-VitD. In the mentioned study, the mean VitD serum levels in the control, CRSwNP, and CRSsNP groups were 51±4.9, 18±4, and 45±2 ng/ml, respectively. Similar to the present study, VitD serum level in the patients with rhinosinusitis with polyps was significantly lower than that in the healthy participants. The lack of difference in the serum VitD levels between the control and CRSsNP groups may be due to the small sample size of their study. The main limitation of the mentioned study is its small sample size, making it difficult to draw conclusions (17).

## Conclusion

As the findings of the present study indicated, serum level of 25-OH-VitD was significantly lower in CRS patients, compared with that in the non-CRS subjects. The usual problem with the investigation of patients with CRS is that we are not faced with a similar group of patients. Regarding this, it is recommended to perform similar studies with a larger sample size and divide patients with CRS into various groups rather than only two groups with and without nasal polyposis, such as fungal CRS and fungal allergic CRS. 

Furthermore, the results of this study can set the ground for planning future studies to investigate the exact relationship between the serum level of VitD and CRS. It is proposed to examine the association between different serum levels of VitD and severity of CRS. It is also recommended to study the effects of VitD levels on nasal mucosa at cellular level. Moreover, it is suggested to design randomized control trials to investigate the remedial effects of VitD in patients with CRT.
